# Attenuation of High Glucose-Induced Damage in RPE Cells through p38 MAPK Signaling Pathway Inhibition

**DOI:** 10.3389/fphar.2021.684680

**Published:** 2021-05-07

**Authors:** Grazia Maugeri, Claudio Bucolo, Filippo Drago, Settimio Rossi, Michelino Di Rosa, Rosa Imbesi, Velia D’Agata, Salvatore Giunta

**Affiliations:** ^1^ Section of Anatomy, Histology and Movement Sciences, Department of Biomedical and Biotechnological Sciences, University of Catania, Catania, Italy; ^2^ Pharmacology Section, Department of Biomedical and Biotechnological Sciences, University of Catania, Catania, Italy; ^3^ Center for Research in Ocular Pharmacology (CERFO), University of Catania, Catania, Italy; ^4^ Eye Clinic, Multidisciplinary Department of Medical, Surgical and Dental Sciences, University of Campania “Luigi Vanvitelli”, Napoli, Italy

**Keywords:** retinal pigment epithelial cells, diabetic retinopathy, VEGF, dimethyl fumarate, p38 MAPK, retina

## Abstract

This study aimed to investigate the high glucose damage on human retinal pigment epithelial (RPE) cells, the role of p38 MAPK signaling pathway and how dimethyl fumarate can regulate that. We carried out *in vitro* studies on ARPE-19 cells exposed to physiological and high glucose (HG) conditions, to evaluate the effects of DMF on cell viability, apoptosis, and expression of inflammatory and angiogenic biomarkers such as COX-2, iNOS, IL-1β, and VEGF. Our data have demonstrated that DMF treatment attenuated HG-induced apoptosis, as confirmed by reduction of BAX/Bcl-2 ratio. Furthermore, in RPE cells exposed to HG we observed a significant increase of iNOS, COX-2, and IL-1β expression, that was reverted by DMF treatment. Moreover, DMF reduced the VEGF levels elicited by HG, inhibiting p38 MAPK signaling pathway. The present study demonstrated that DMF provides a remarkable protection against high glucose-induced damage in RPE cells through p38 MAPK inhibition and the subsequent down-regulation of VEGF levels, suggesting that DMF is a small molecule that represents a good candidate for diabetic retinopathy treatment and warrants further *in vivo* and clinical evaluation.

## Introduction

Diabetic retinopathy (DR) is the leading cause of visual impairment and preventable blindness, and it represents an important cost in terms of social and economic issues for health care systems worldwide. Vision loss is associated with retinal pigment epithelial (RPE) damage, these retinal cells play a key role in terms of protection and functional maintenance of photoreceptors (Plafker et al., 2012). Pre-clinical studies and clinical trials showed that hyperglycemia represents the primary risk factor leading to DR (Cade, 2008). It has been observed that sustained hyperglycemia promotes the activation of both apoptotic (Kowluru et al., 2004) and inflammatory mechanisms (Yuuki et al., 2001) as well as dysregulation of growth factors and hypoxia-inducible factors (Grant et al., 2004; Schlingemann, 2004; D’Amico et al., 2015). Upregulation of cytokines and other proinflammatory mediators inducing chronic inflammation, is believed to actively contribute to the DR-associated damage to the retinal vasculature (Zheng and Kern, 2009; Boss et al., 2017) also by triggering apoptosis of RPE cells and promotion of retinal neovascularization (Kim et al., 2015; Xiao et al., 2017).

The vascular endothelial growth factor (VEGF) triggers the angiogenesis modulating vascular permeability, and it is hypothesized as the key factor in the pathogenesis of DR and diabetic macular edema (Giurdanella et al., 2015; Platania et al., 2015; Bolinger and Antonetti, 2016; Bucolo et al., 2018). The expression of VEGF by RPE cells has been analyzed in different studies, and the results have contributed to our knowledge on the involvement RPE cells in the pathophysiology of DR (Saishin et al., 2003; Campochiaro, 2013). Elevated VEGF levels were obtained in vitreous, aqueous, and subretinal fluid of patients with DR and other retinal disorders, even though VEGF levels were different in patients affected by proliferative and non-proliferative DR (Aiello et al., 1994; Rubio and Adamis, 2016). Angiogenesis and inflammation are two important components implicated in the pathogenesis of DR (Bucolo et al., 2013; D’Amico et al., 2015; Platania et al., 2017). Recent studies provided evidence that dimethyl-fumarate (DMF) has a strong anti-inflammatory action, although the effect of DMF on angiogenesis is unknown. Interestingly, Zhao et al. (2014) has demonstrated that DMF can affect the release of VEGF.

DMF is an efficient immunomodulatory drug, approved in clinical practice for the treatment of relapsing–remitting multiple sclerosis and psoriasis. The primary pharmacodynamic response to DMF treatment is activation of HO-1 gene through the regulation of nuclear factor erythroid-2–related factor-2 (Nrf2). However, DMF seems to have several biological effects as it has been shown to also inhibit the phosphorylated p38 MAPK (Nishioku et al., 2020; Kortam et al., 2021) thus blocking downstream targets that may be involved in the development and progression of inflammatory cascades leading to numerous diseases. In fact, it has been demonstrated that the activation of p38 MAPK signaling is required for VEGF expression in response to growth factors and cytokines stimulation (Gomes and Rockwell, 2008; Sakai et al., 2017).

In the present study we explored the hypothesis that the DMF is able to interfere with p38 MAPK signaling and consequently down-regulate VEGF levels in RPE cells exposed to high glucose damage. To address this issue, we carried out *in vitro* studies on human RPE cells exposed to normal (NG) or high (HG) glucose conditions, in order to figure out the effects of DMF on cell viability, apoptosis and expression of inflammatory and angiogenic biomarkers such as COX-2, iNOS, IL-1β, and VEGF.

## Materials and Methods

### Cells

This study was performed on human retinal pigment epithelial cell culture (ARPE-19) purchased from the American Type Culture Collection (Rockville, United States). Cells cultured in DMEM:F12 medium containing 10% fetal bovine serum (FBS), 100 U/ml penicillin, 10 μg/ml streptomycin, were incubated at 37°C in a humidified atmosphere with 5% CO_2_. Once cells reached confluence, they were exposed to normal glucose condition (NG, 5.5 mM D-glucose) or to high glucose (HG, 25 mM D-glucose) in order to mimic physiological or hyperglycemic conditions, respectively (Maugeri et al., 2017). To exclude any potential bias by an osmotic effect, a separate control group of cells were also grown in NG medium plus mannitol (5.5 mM D-glucose and 19.5 mM mannitol vs. 25mM D-glucose). All experiments and measurements described below were performed in parallel in NG and HG conditions.

### Chemicals and Reagents

Dimethyl fumarate (DMF) was obtained by Sigma-Aldrich (St. Louis, MO, United States). A selective p38 MAP kinase inhibitor SB202190 was purchased from Abcam (Cambridge, United Kingdom). All other chemicals and reagents were purchased by Sigma-Aldrich unless otherwise stated. The stock solution of DMF was prepared using DMSO and diluted with cell culture medium to obtain final dose for each treatment. As indicated, vehicle (0.2% v/v DMSO) corresponds to the amount present in the highest dosage and was used in all experiments to exclude unspecific and toxic effects.

### Cell Viability

Cell viability was measured using the cell proliferation kit I (MTT) according to manufacturer’s procedures (Roche, Basel, Switzerland) (Maugeri et al., 2018a). Cells were seeded into 96-well plates at a density of 1 × 10^4^ cells/well. After the treatments, medium containing 0.5 mg/ml 3-[4,5-dimethylthiazol-2-yl]-2,5-diphenyltetrazolium bromide (MTT) (Sigma Aldrich, St. Louis, MO, United States) was added in each well. Following incubation for 4 h at 37°C, medium was removed, and 100 μL of DMSO was added. Formazan generated by the cleavage of the yellow tetrazolium salt MTT was measured at 570 nm using a microplate reader (BioRad Laboratories, Milan, Italy).

### Apoptotis

The effects of HG and DMF on apoptosis and nuclear morphology in the cells were assessed by the use of fluorescence microscopy with the nuclear dye Hoechst 33,258 as previously described (Bucolo et al., 2019). After indicated treatment, cells were fixed with a solution of methanol/acetic acid (3:1 v/v) for 30 min, washed with ice-cold PBS and stained for 15 min at 37°C with 0.4 μg/ml Hoechst 33,258 dye. After being rinsed in water, cells were observed under an Axiovert 40 fluorescence microscope (Carl Zeiss Inc., Thornwood, NY, United States). Apoptosis and nuclear morphology were identified by condensation of nuclear chromatin and its fragmentation. Each condition was reproduced in three dishes per experiment. Both apoptotic and normal cells were determined by analyzing at least three different fields per dish in a fixed pattern.

### ELISA

IL-1ß and VEGF levels were assessed by ELISA analysis, performed in fresh supernatant derived from serum-free media of cells cultured with or without treatments. Briefly, cell supernatant was collected through centrifugation (500 Å∼ g, 5 min, 4°C). The levels of IL-1β and VEGF in the supernatant (100 μL) of ARPE-19 cells were detected using sandwich ELISA kits according to the manufacturer’s protocol of each kit (DY201, R&D Systems, Minneapolis, United States, and ENZ-KIT156-0001, Enzo Life Science, Milano, Italy, respectively).

### Western Blot

Protein extracts were detected by western blot as previously described by D’Amico et al. (2018). Primary antibodies: BAX (sc-493, Santa Cruz Biotechnology, Inc, CA, United States), Bcl-2 (sc-509, Santa Cruz Biotechnology, Inc, CA, United States), COX-2 (sc-19999, Santa Cruz Biotechnology, Inc, CA, United States), iNOS (sc-651, Santa Cruz Biotechnology, Inc, CA, United States), p38 MAPK (#9212, Cell Signaling Technology, United States), p-p38 MAPK (Thr180/Tyr182) (#9211, Cell Signaling Technology, United States) and *β*-tubulin (sc-9104, Santa Cruz Biotechnology, Inc, CA, United States). Secondary antibodies: goat anti-Rabbit IRDye 680 (cat #926-68021, LI-COR Biosciences, Lincoln, NE, United States) and goat anti-Mouse IRDye 800CW (cat #926-32210, LI-COR Biosciences, Lincoln, NE, United States).

### Immunolocalization

The cellular distribution of VEGF was detected by immunofluorescence assay as described in a previous study (Maugeri et al., 2018b). Cells cultured on slides, were rinsed with PBS and then fixed in 4% paraformaldehyde in PBS, permeabilized with 0.2% Triton X100, blocked with 0.1% BSA in PBS, and then incubated with anti-VEGFA antibody (ab1316, Abcam, Cambridge United Kingdom). The fixed cells were then washed, and Alexa Fluor 594-conjugated secondary antibodies (Thermo, United States) were used to stain the corresponding primary antibodies for 1.5 h at 37°C. After a sequence of washes, the fixed cells were cover-slipped with vecta-shield mounting medium (Vector Laboratories, Inc, Burlingame, CA, United States). Ten fields from randomly selected slides were visualized using an Axiovert 40 epifluorescence microscope (Carl Zeiss Inc, Thornwood, NY, United States) at ×40 magnification and images of each field were captured using a digital camera (Canon, Ōta, Tokyo, Japan).

### Semi-Quantitative Reverse Transcription–Polymerase Chain Reaction (RT-PCR)

Semi-quantitative RT-PCR was performed as described in detail in an earlier report (Castorina et al., 2010). Total cellular RNA was isolated through the use of 1 ml TRIzol reagent (Invitrogen), 0.2 ml chloroform and precipitated with 0.5 ml isopropanol. Aliquots of cDNA were amplified using specific primers matching the reported sequence of human VEGF and ribosomal protein S18 (RPS18), used as reference gene. The primers used were the following: VEGF (forward: 5′-GAA​GTG​GTG​AAG​TTC​ATG​GA-3′; reverse: 3′-GCC​TTG​CAA​CGC​GAG​TCT​GT-5′) and RPS18 (forward: 5′-GAG​GAT​GAG​GTG​GAA​CGT​GT-3′; reverse: 3′-GGA​CCT​GGC​TGT​ATT​TTC​CA-5′). Semi-quantification of the density of each band was done by ImageJ software (US National Institutes of Health).

### Statistical Analysis

GraphPad InStat version 3.00 (GraphPad Software Inc, San Diego CA, United States) was used to analyze the experimental data. All the data are expressed as mean ± SEM. The data from multiple groups were analyzed by one-way ANOVA and statistical significance was assessed by Tukey-Kramer post-hoc test, unless otherwise indicated. The level of significance accepted for all statistical tests was *p* ≤ 0.05.

## Results

### Anti-Apoptotic Effect of DMF on RPE Cells Exposed to High Glucose

**FIGURE 1:**
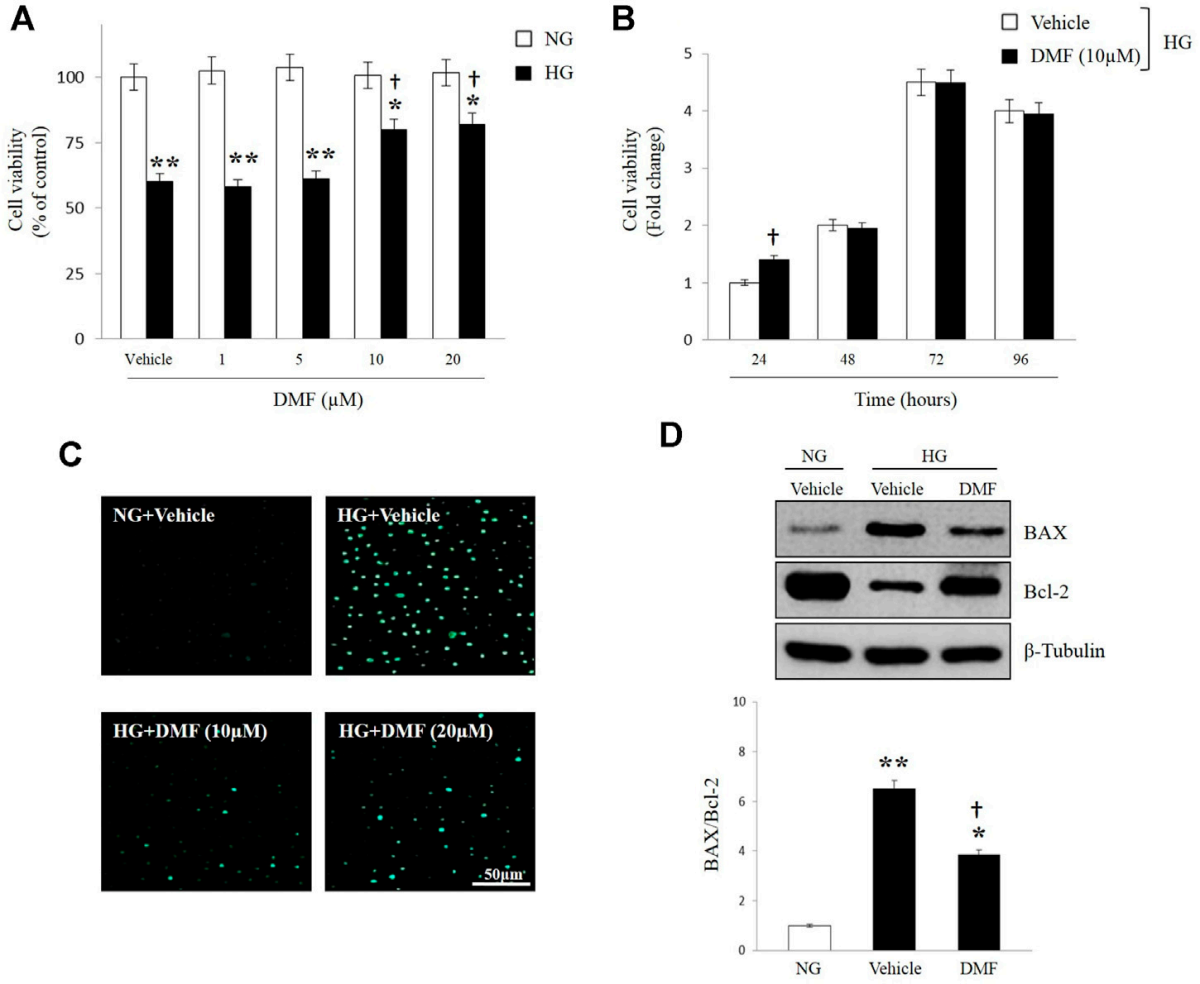
Effects of DMF on RPE cells viability. ARPE-19 cells exposed to normal glucose (NG) or high glucose (HG) w/or w/o DMF. Cell viability **(A,B)**, DNA damage **(C)** BAX and Bcl-2 protein expression **(D)** were assessed. **(A,B)** MTT analyses: values are expressed as percentage of cell viability of NG treated-vehicle group (Vehicle/NG) or Fold change ± SEM. (*n* = 6; *p < 0.05 or **p < 0.01 vs. Vehicle/NG; †*p* < 0.05 vs. Vehicle/HG. **(C)** Apoptotic assay by nuclear staining: Representative photomicrographs of ARPE cells exposed to NG and HG and treated with 10 or 20 μM DMF up to 24 h. Cells were stained with the fluorescent nuclear dye Hoechst 33,258 and observed at ×40 magnification. Scale bar = 50 μm. (**D**) Western blot analyses of BAX and Bcl-2 in ARPE cells exposed to HG w/or w/o DMF (10 μM) for 24 h. Band densities were quantified by ImageQuantTL software and normalized values were indicated below each corresponding band. Normalized band density values in control groups (NG) were set to 1. *p < 0.05 or **p < 0.01 vs. NG; †*p* < 0.01 vs. Vehicle/HG; *n* = 6.

DMF effect on human RPE cells survival exposed to HG insult, was evaluated through MTT assay. ARPE-19 cells were cultured in NG or HG and exposed to different concentration of DMF (1, 5, 10, and 20 μM) for 24 h. As shown in Figure 1A, HG exposure significantly reduced cells viability as compared to cells grown in NG (*p* < 0.01 vs. Vehicle/NG). Treatment with DMF attenuated this effect in a dose-dependent manner with significant values at concentrations of 10 and 20 µM (*p* < 0.05 vs. Vehicle/HG). The effect of DMF was also studied in a time-dependent manner. ARPE-19 cells were incubated for 24, 48, 72, and 96 h in HG condition and treated with 10 µM of DMF. As shown in Figure 1B, DMF significantly increased cells viability at 24 h as compared to vehicle (*p* < 0.05 vs. Vehicle/HG).

In order to evaluate if high glucose levels induced the activation of apoptotic process and to investigate the possible protective role of DMF, ARPE-19 cells were exposed to NG or HG with or without DMF and assayed for DNA fragmentation. Representative images in Figure 1C showed the morphological signs of nuclear damage (as determined by Hoechst 33,258 staining) in response to HG exposure for 24 h. On the contrary, cells cultured under high glucose insult and treated with 10 or 20 µM of DMF generated only slight DNA damage, which was greatest at 10 µM of concentration. The anti-apoptotic effect of DMF was confirmed by western blot analysis. Figure 1D showed that BAX (proapoptotic)/Bcl-2 (antiapoptotic) ratio was significantly increased in HG exposed cells as compared to ARPE-19 cultured in NG (*p* < 0.05 or *p* < 0.01 vs. NG). As expected, DMF treatment significantly reduced BAX/Bcl-2 ratio (*p* < 0.05 vs. Vehicle/HG).

### Effect of DMF on Inflammatory Process of RPE Cells

**FIGURE 2:**
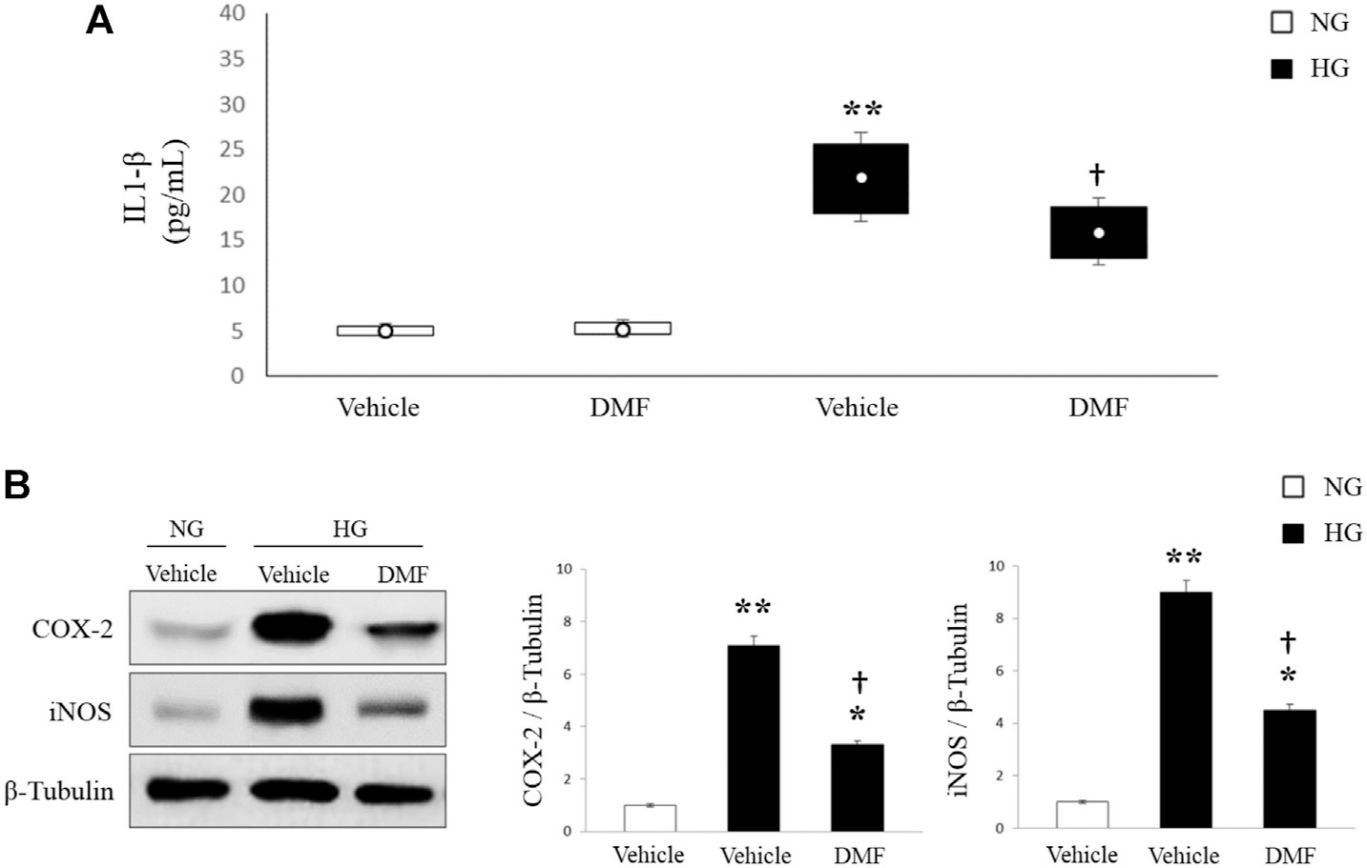
Effects of DMF on inflammatory cascade of ARPE-19 cells. ARPE-19 cells exposed to normal glucose (NG) or high glucose (HG) w/or w/o DMF for 24 h. **(A**) IL-1ß production was measured through ELISA. (*n* = 6, **p < 0.01 vs. Vehicle/NG; †*p* < 0.05 vs. Vehicle/HG). (**B**) Western blot: COX-2 and iNOS expression proteins. Band densities were quantified by ImageQuantTL software and normalized values were indicated below each corresponding band. *p < 0.05 or **p < 0.01 vs. Vehicle/NG; †*p* < 0.05 vs. Vehicle/HG; *n* = 6.

Increase of inflammatory process and production of pro-inflammatory cytokines was largely demonstrated in the vitreous of diabetic patients, in the retina of STZ-induced diabetic rats and also in the RPE cells under high glucose conditions. According with these findings (Figure 2A) we found high levels of IL-1ß in ARPE-19 cells exposed to HG (*p* < 0.01 vs. Vehicle/NG). Interestingly, treatment of HG-cultured cells with DMF (10 µM) significantly reduced the release of this pro-inflammatory cytokine (*p* < 0.05 vs. Vehicle/HG).

Considering that IL-1ß is a trigger of the neuroinflammatory cascade stimulating COX enzymes and iNOS, we analyzed through western blot the expression levels of iNOS and COX-2 proteins. As shown in Figure 2B, iNOS and COX-2 expression levels were significantly increased in ARPE-19 cells grown in HG as compared to NG cultured cells (*p* < 0.01 vs. NG). Interestingly, treatment with 10 µM of DMF significantly reduced the expression of both inflammatory mediators (*p* < 0.05 vs. Vehicle/HG).

### Effect of DMF on VEGF Expression in ARPE-19 Cells

**FIGURE 3:**
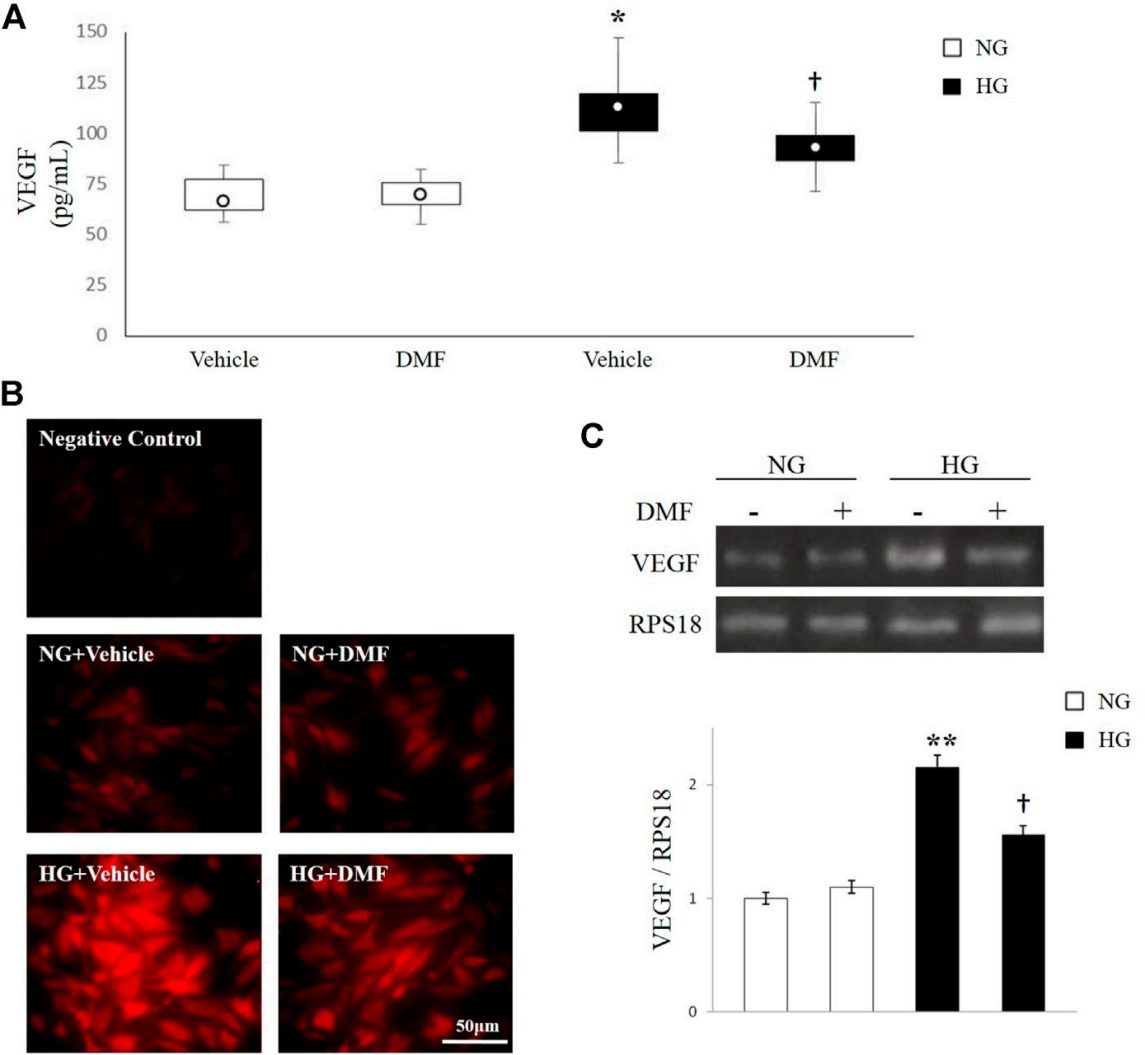
Effects of DMF on VEGF levels. ARPE-19 cells exposed to normal glucose (NG) or high glucose (HG) w/or w/o DMF (10 μM) for 24 h. **(A)** VEGF production was measured through ELISA. *p < 0.05 vs. Vehicle/NG; †*p* < 0.05 vs*.* Vehicle/HG; *n* = 6. **(B)** Representative photomicrographs showing VEGF expression (red). Photomicrographs are representative results of fields taken randomly from each slide and observed by Axiovert 40 epifluorescence microscope (magnification ×40). **(C)** Semi-quantitative RT-PCR shows VEGF mRNA expression normalized to the ribosomal protein S18 (RPS18, housekeeping gene). **p < 0.01 vs. NG untreated cells, †*p* < 0.05 vs. untreated/HG; *n* = 6.

It is well known that hyperglycemia triggers angiogenesis in RPE cells. Here, we evaluated the effect of DMF on VEGF production in ARPE-19 cells exposed to high glucose insult through ELISA, immunofluorescence analysis and semi-quantitative RT-PCR. As shown in Figure 3A, the levels of VEGF in the culture media of ARPE-19 cells exposed to high glucose conditions significantly increased in comparison with NG exposed cells (*p* < 0.05 vs. Vehicle/NG). Conversely, DMF treatment significantly reduced the production of VEGF (*p* < 0.05 vs. Vehicle/HG). These data were also confirmed through immunofluorescence analysis. In fact, as shown in Figure 3B, VEGF was weakly expressed in NG with or without DMF treatment. On the contrary, HG exposure induced a dramatic increase of VEGF expression in ARPE-19; this effect was significantly attenuated by DMF treatment.

The reduction of VEGF levels mediated by DMF treatment was further demonstrated by analyzing the expression of VEGF mRNA through semiquantitative RT-PCR, by using specific primers recognizing the angiogenic factor. Consistent with the results of ELISA and immunofluorescence analysis, the mRNA levels of VEGF were significantly increased after HG stimulation compared to NG grown cells (*p* < 0.01 vs. NG). As expected, DMF partially decreased VEGF expression (*p* < 0.05 vs. Vehicle/HG).

### Effects of DMF on p38 MAPK Activation

**FIGURE 4:**
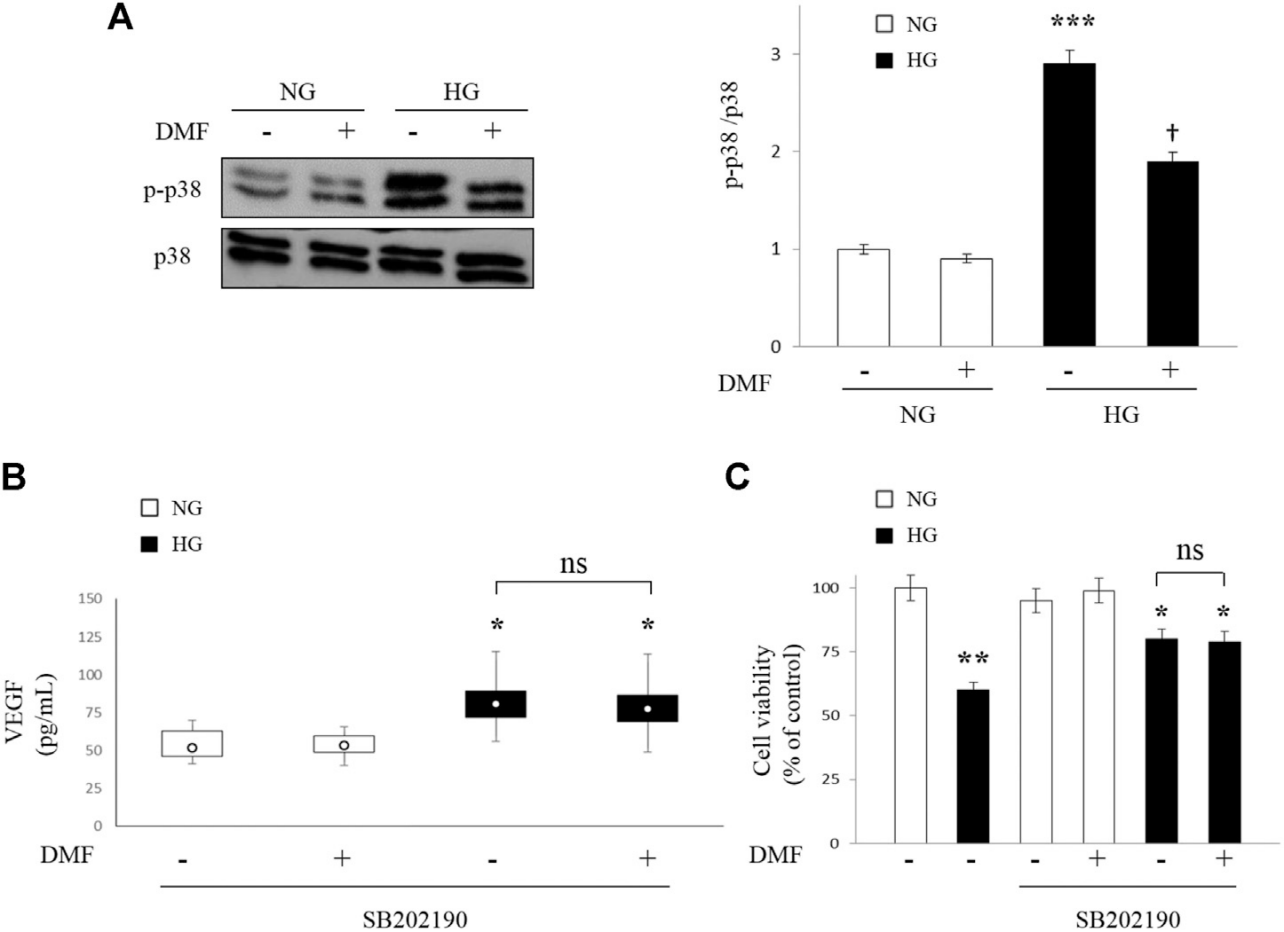
Effects of DMF on p38 MAPK pathway. ARPE-19 cells exposed to normal glucose (NG) or high glucose (HG) w/or w/o DMF (10 μM) for 24 h **(A)** p38 MAPK was measured by western blot analysis; band densities were quantified by ImageQuantTL software and normalized values were plotted in the histogram shown on the right (***p < 0.001 vs. NG; †*p* < 0.05 vs. untreated/HG; *n* = 6). **(B)** ARPE-19 cells exposed to normal glucose (NG) or high glucose (HG) were pretreated with SB202190 (10 µM), a p38 MAPK inhibitor for 30 min and then treated with DMF (10 μM) for 24 h. VEGF levels were measured through ELISA. *p < 0.05 vs. NG untreated cells; *n* = 6. (**C**) MTT analysis: values are expressed as percentage of cell viability in NG untreated cells. *p < 0.05 or **p < 0.01 vs. NG untreated cells; *n* = 6.

In order to investigate the molecular mechanisms involved in DMF protection, we have analyzed the activation of p38 MAPK signaling cascades, given that literature shown that activation of this signaling pathway affect the expression of VEGF. To address this issue, we performed western blot analyses to assess any changes in p38 MAPK protein levels as well as in its phosphorylated forms in ARPE-19 cells exposed to NG or HG conditions with or without DMF (10 µM) up to 24 h. The results of Figure 4A indicated that HG exposure significantly increased the phosphorylation of p38 MAPK levels (*p* < 0.001 vs. NG). Interestingly, DMF administration partially counteracted HG-induced p38 MAPK activation (*p* < 0.05 vs. untreated/HG).

ARPE-19 cells, both in NG and HG conditions, were pre-treated with the specific p38 MAPK inhibitor (SB202190, 10 µM) for 30 min prior to DMF treatment for 24 h and VEGF production (ELISA) and cell viability (MTT analysis) were measured. We chose these experimental conditions based on previous data (Saika et al., 2005; Cheng et al., 2019). As shown in Figure 4B, the previously observed HG-induced VEGF increase (see Figure 3A) were partially reversed by SB202190 pre-treatment and, more important, any significant differences in the levels of VEGF were detected in HG grown cells after DMF administration (n.s. vs. untreated/HG), confirming that the protective effect of DMF is attributable to p38 MAPK pathway inhibition.

We confirmed that the effect of DMF is attributable to p38 MAPK pathway inhibition through MTT assay. In fact, as shown in Figure 4C, no significant difference was observed between cells pre-treated with SB202190 with or without DMF (n.s. vs. DMF-untreated/HG).

## Discussion

In the present study, we showed that DMF counteracts high glucose-induced damage in RPE cells through the attenuation of inflammatory cascades that involves p38 MAPK pathway-mediated VEGF inhibition suggesting a protective role in the pathologic events associated to hyperglycemia. The protective role of DMF in nervous system has been extensively demonstrated in several paradigms of neuroinflammation both *in vitro* and *in vivo*. In particular, DMF counteracted the apoptotic cascade by increasing cells survival and reducing the inflammatory process (Wilms et al., 2010; Linker et al., 2011; Scannevin et al., 2012; Wang et al., 2015). The beneficial role of DMF was also demonstrated in the retina (Cho et al., 2015). In particular, DMF down-regulated inflammatory genes expression, counteracted reactive Müller cell gliosis and reduced cell loss by promoting the physiological functions of the retina against ischemia-reperfusion injury (Cho et al., 2015). Moreover, DMF protected retinal tissue in streptozotocin-induced diabetic rats (Patil et al., 2008; Fan et al., 2012). On this regards it could be useful develop in the future a biodegradable deliver system to administer DMF into the eye (Conti et al., 1997). Recently, antioxidant and wound healing properties of DMF were demonstrated in RPE cells challenged with high glucose (Foresti et al., 2015). Based on these previous results, the present study was designed to further confirm the protective effects exerted by DMF on outer BRB during HG exposure. It is well known that outer BRB is impaired under sustained hyperglycemia, and RPE cell loss is a key event in the progression of DR (Chen et al., 2016; Xia and Rizzolo, 2017). According with these data, we observed a dramatic reduction of RPE cells viability, up to 50%, compared to RPE cells exposed to physiological levels of glucose (5mM, NG). Interestingly, DMF treatment protects RPE cells exposed to high glucose, maintaining their viability in a dose-dependent manner. Further, we showed that DMF decreased the expression of apoptotic gene (Bax) and increased the expression of anti-apoptotic genes (Bcl-2) in RPE cells. This result leads to a decrease in the ratio of pro-apoptotic Bax/anti-apoptotic Bcl2 genes, which causes apoptosis reduction. These observations suggest that DMF has the potential to attenuate apoptotic events associated to hyperglycemia. The HG-induced damage to RPE layer is also due to a severe inflammation sustained by the release of inflammatory cytokines including IL-1β (Joussen et al., 2004; Kern, 2007; Wang et al., 2019). IL-1ß is known to induce vascular dysfunction as well as apoptosis cell death during DR progression (Vincent and Mohr, 2007; Zhang et al., 2019). The immunomodulation role of DMF has already been reported in literature; in fact, previous studies displayed that DMF increases anti-inflammatory cytokines and reduces the pro-inflammatory cytokines like tumor necrosis factor-alpha, IL-2 and IL-17 (Albrecht et al., 2012). In the present study, we showed that DMF treatment decreased the IL-1β levels in HG-cultured RPE cells. The evidence that DMF plays an anti-inflammatory role was further supported by the analysis of COX-2 and iNOS, two inflammatory markers over-expressed by different retinal cell types in response to high levels of glucose (Du et al., 2013; Shin et al., 2014). The present findings showed a significant decrease expression of COX-2 and iNOS in RPE exposed to high glucose when treated with DMF.

It has been demonstrated that the hyperglycemic insult promotes release and activation of the VEGF (Yancopoulos et al., 2000) and RPE cells exposed to high glucose synthetized high levels of this angiogenic factor (Sone et al., 1996). Here, we have shown for the first time that DMF attenuates expression of VEGF mRNA and its release from RPE cells exposed to HG. Since the p38 MAPK pathway promotes and regulates the expression of pro-angiogenesis factors, including VEGF (Gomes and Rockwell, 2008; Sakai et al., 2017) we sought to determine whether VEGF decrease could rely on a DMF-induced p38 MAPK down-regulation. Therefore, we performed Western blot and ELISA assay to further explore the role of DMF on the modulation of p38 MAPK pathway and VEGF levels production. Our results showed that the expression of phosphorylated form of p38 MAPK, was significantly down-regulated by DMF in HG exposed cells. The inhibitory effect of DMF on p38 MAPK phosphorylation was consistent with other studies in human cells stimulated with IL-8 and tumor necrosis factor-α (Nishioku et al., 2020; Kortam et al., 2021). Moreover, the SB202190 reduced the release of VEGF at comparable levels to reduction induced by DMF, and co-treatment of SB202190 with DMF did not alter VEGF secretion.

## Conclusion

The present data demonstrated that DMF provides protection against high glucose-induced damage in human RPE cells counteracting inflammation and angiogenesis *via* p38 MAPK/VEGF pathway. In conclusion these findings suggested that DMF represents a potential good candidate for diabetic retinopathy treatment and warrants further *in vivo* and clinical evaluation.

## Data Availability

The raw data supporting the conclusions of this article will be made available by the authors, without undue reservation.
